# Non-extraction Approach in a Borderline Case of a Growing Patient: A Case Report

**DOI:** 10.7759/cureus.62195

**Published:** 2024-06-11

**Authors:** Trupti Chikankar, Japneet Kaiser, Khyati Gupta, Ranjit Kamble

**Affiliations:** 1 Department of Orthodontics and Dentofacial Orthopedics, Sharad Pawar Dental College and Hospital, Datta Meghe Institute of Higher Education and Research, Wardha, IND

**Keywords:** non-extraction, orthodontics, retainers, malocclusion, crowding

## Abstract

This case presents the effective non-extraction orthodontic treatment of a 13-year-old boy with crowding in both upper and lower arches and deep bite. The patient's chief complaint was irregularly placed maxillary anterior teeth. The active treatment duration lasted for 10 months, which resulted in the successful alleviation of arch crowding and correction of the deep bite without the extraction of any sound erupted tooth. Posttreatment, all of the patient's chief complaints were relieved. Essix retainers were fitted post-debonding, with instructions for the patient to wear them for the subsequent year to maintain the achieved results. This case highlights the efficacy of non-extraction orthodontic strategies in addressing crowding and deep bite issues, drawing the importance of individualized treatment plans to achieve optimal outcomes.

## Introduction

A discrepancy between maxillary and mandibular arches in any of the dimensions or the existence of irregularities in tooth position is referred to as malocclusion [1]. It is regarded as a condition wherein oral health and periodontal health are affected. It raises concerns related to facial esthetic and psychosocial problems and can cause clicking and pain in the temporomandibular joint or other serious risks to overall oral health [1,2]. In the last decade, orthodontic corrections for esthetic purposes have risen significantly. The alignment of the anterior teeth is specifically important as they are the first to be noticed in a smile. Proper orthodontic treatment enhances occlusion and facial esthetics. Thus, the majority of people look for orthodontic treatment [3]. Malocclusions are classified into Classes I, II, and III according to the molar relationship [4]. Because of the presence of Angle Class I malocclusion, around 61% of children in the age group 13-15 years need orthodontic correction [5]. Angle’s Class I malocclusion was described as occlusion of the mesiobuccal cusp of the upper first molar with the mesiobuccal groove of the lower first molar [6]. The characteristics of Class I malocclusion could be a deep bite, crowding, spacing, crossbite (unilateral or bilateral), and an anterior open bite. Among all these features, crowding is frequently seen in Class I malocclusion [7]. Crowding is seen when there is a disproportion between the arch and teeth sizes [8]. In a crowded malocclusion, teeth tend to erupt labial or lingual to the line of the arch [7].

The treatment for crowding is either resolved by extraction of teeth in both arches or by no extraction [9]. Both of these approaches have their own set of benefits and setbacks, which is why choosing a favorable option depending on the age of the patient, the degree of crowding, and the jaw that is impacted can influence the treatment outcome [9]. The conflict of choosing either of the methods has been going on for the longest time. In various case studies, it is mentioned that if extraction of teeth is opted, then there is a high chance that it will disrupt the facial harmony and alteration in arch width and form post-extraction and can cause abnormal function [10]. With a non-extraction approach, facial harmony can be achieved, it is a less invasive procedure, and the treatment and recovery time is significantly reduced. With a non-extraction approach, crowding can be resolved by the advancement of anterior teeth, posterior teeth distalization, and arch expansion transversely [11,12].

This case narrates the orthodontic treatment without extraction of a healthy tooth in an Angle's Class I malocclusion in which the patient complains of upper arch crowding.

## Case presentation

**Figure 1:**
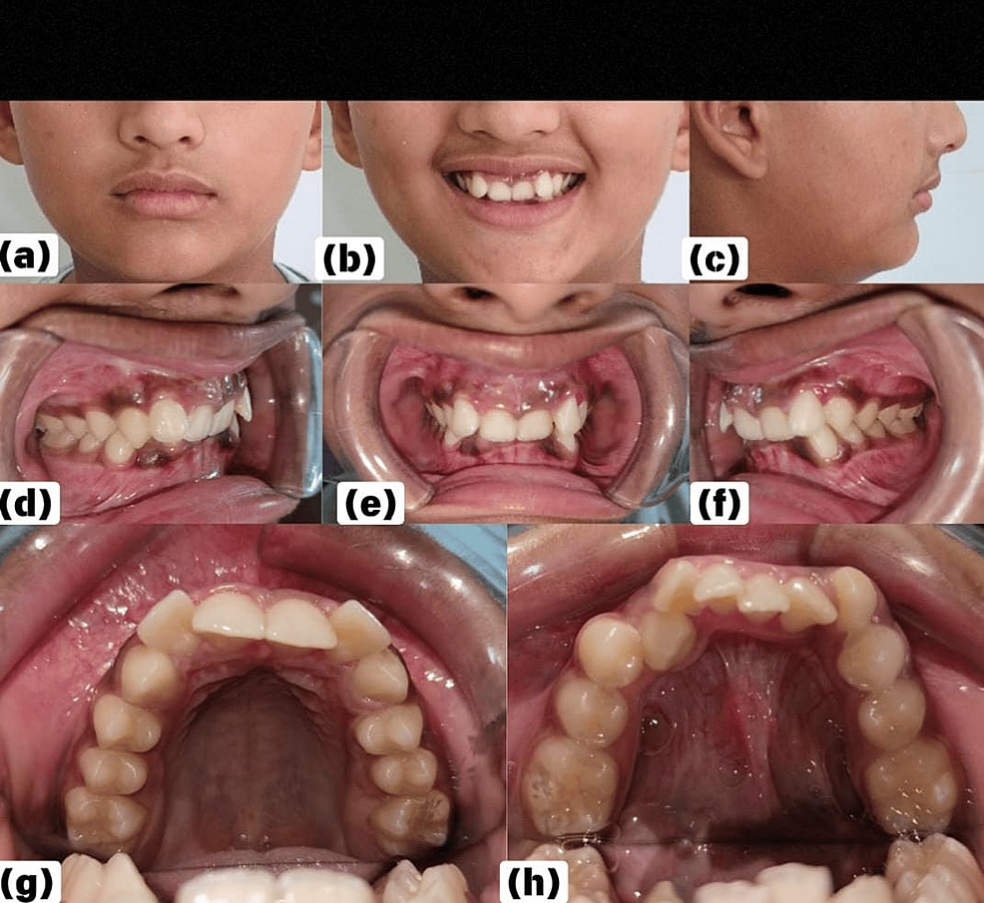
Pretreatment facial and intraoral photographs (a) Frontal, (b) smiling, (c) side profile, (d) occlusion on the right side, (e) frontal view of occlusion showing crowding and deep bite, (f) occlusion on the left side, (g) maxillary crowding, (h) mandibular crowding

A healthy 13-year-old male reported to the Department of Orthodontics with a chief complaint of misaligned front teeth in the upper and lower arches. The patient reported that he had never undergone any orthodontic correction previously and wanted to align his irregularly placed teeth. An extraoral examination revealed competent lips, symmetrical face, convex face profile, mesocephalic head shape, deep submental fold, and gummy smile from the frontal view as observed in Figures 1a-1c and normal speech function. An intraoral examination revealed Angle's Class I malocclusion on both the left and right sides (Figures 1d, 1f). In the maxilla, there was retroclination of the central incisors and proclination of the lateral incisors. The crowding of 5 mm in the anterior teeth was measured. The palatal vault was deep, and the maxillary arch appeared square because of retroclined central incisors. In the mandible, crowding of 5 mm was seen in the anterior teeth. The canines on either side were slightly rotated (Figures 1g-1h). The midline of the maxillary and mandibular arch coincided with each other and with the facial midline. The overbite was 7 mm, and the overjet was 1 mm. A complete deep bite was present, as seen in Figures 1d-1f. The overall health of the teeth and periodontium was satisfactory. Analysis of the dental cast revealed crowding in both arches. The arch discrepancy in the maxilla was 3 mm and in the mandible 5-6 mm.

**Figure 2:**
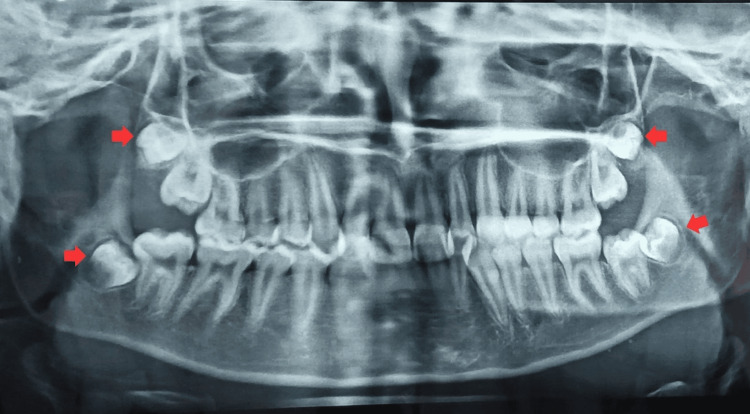
An orthopantomogram showing developing third molars in both maxilla and mandible

**Figure 3:**
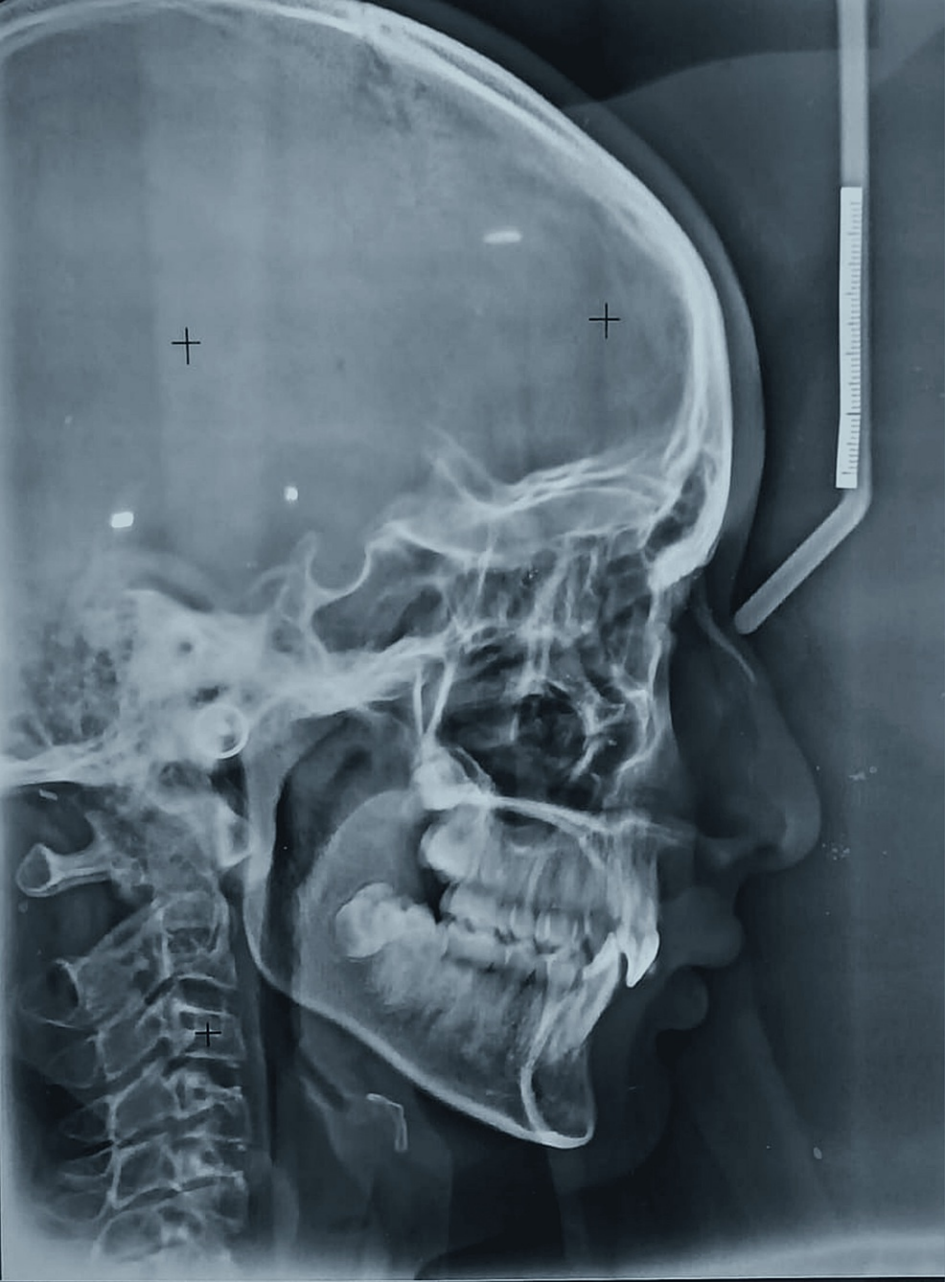
Lateral cephalogram

In panoramic radiographs, all third molars were observed in developmental stages. No pathological finding was detected (Figure 2). Lateral cephalometric analysis was done using Down's, Steiner's, and Tweed's analysis. The analysis values in Table 1 depict skeletal Class II with an average growth pattern, slightly retruded mandible, and dentoalveolar Class I with Dewey’s type I modification. It revealed the retroclined of the maxillary anterior with 1-NA value to be 2 mm and an angle of 10°. Lower incisors were also retroclined with the 1-NB value of 1 mm and an angle of 10°. The value of the Y axis was 67°, depicting a vertical growth pattern (Figure 3).

**Table 1:** Cephalometric analysis SN: sella-nasion plane; ANB: A point–nasion–B point angle; FMA: Frankfurt mandibular plane angle; FMIA: Frankfurt mandibular incisor plane angle; IMPA: incisor mandibular plane angle; SNA: sella-nasion point A angle; SNB: sella-nasion point B angle; SND: sella-nasion point D angle; NA/NB: nasion to point A/B; Go-Gn: gonion-gnathion, Pog: pognion [13]

Measurements	Mean value	Pretreatment values
SNA	82˚	82°
SNB	80˚	78°
SND	78°	76°
ANB	2°	4°
Go-Gn to SN	32°	28°
Upper incisor to NA (mm)	4 mm	2 mm
Upper incisor to NA (angle)	22˚	10°
Lower incisor to NB (mm)	4 mm	1 mm
Lower incisor to NB (angle)	25˚	10°
Occlusal to SN (angle)	14˚	11°
Facial angle	87.8˚	80°
Angle of facial convexity	0˚	6°
A–B plane angle	-4.8˚	10°
Mandibular plane angle	21.9˚	32°
Y axis	59.4˚	67°
Cant of occlusal plane	9.3˚	13°
Interincisal angle	135.4˚	157°
Lower incisor to occ plane	14.5°	0 mm
Upper incisor to A–Pog	2.7 mm	1
FMA	25°	31°
FMIA	65°	67°
IMPA	90°	82°

Treatment plan

The treatment objectives included correcting the irregularly placed upper and lower anterior dentition by leveling and alignment of teeth, achieving ideal overbite and overjet with the ideal arch form in the upper and lower arches, and maintaining the Class I molar and canine relationship.

Following a comprehensive analysis of clinical examination findings, radiographic evaluation, and diagnostic dental models, the case was diagnosed as Angle's Class I with Deweys’s type 1 modification. A meticulous treatment plan was devised to address the crowding in both the arches and correct anterior deep bite without any tooth extraction. The treatment strategy aimed to harmonize the occlusion and enhance facial esthetics while preserving all healthy erupted teeth. The treatment was initiated with banding of the first molars of both the upper and lower arches. The teeth were bonded using a 0.022-inch slot McLaughlin, Bennett, and Trevisi (MBT) kit, a pre-adjusted edgewise appliance. Further, the treatment progressed with the use of 0.014” Nickel-Titanium (NiTi), and 0.016" NiTi. After the leveling and alignment were complete, 0.016×0.025 NiTi were placed on both arches. This was followed by 0.019×0.025 stainless steel (SS) wire placement for settling the occlusion. There were noticeable changes in both arches during the first postoperative week. Throughout the 10-month active treatment phase, significant improvements were observed (Figures 3a-3h).

**Figure 4:**
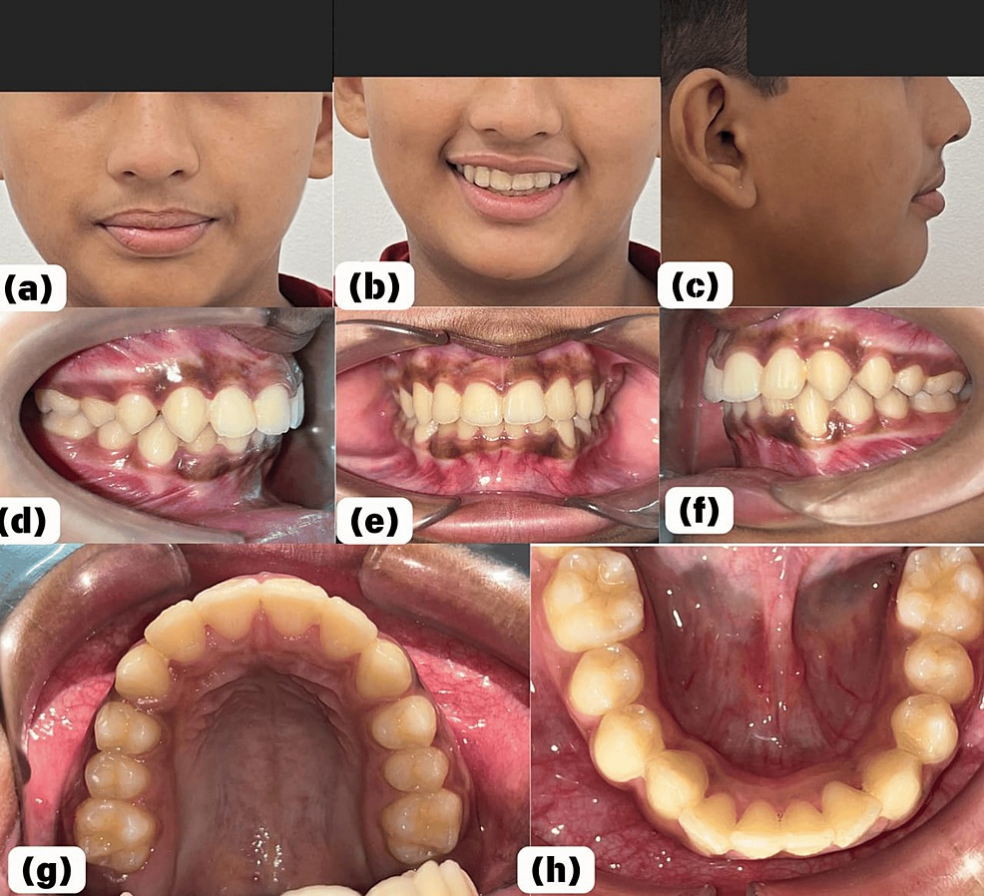
Posttreatment facial and intraoral photographs (a) Frontal view, (b) smiling frontal view, (c) side profile, (d) occlusion on the right side, (e) frontal view showing corrected anterior crowding and deep bite, (f) occlusion on the left side, (g) corrected maxillary crowding without extraction, (h) corrected mandibular crowding without extraction

After 10 months of treatment, debonding was done. Posttreatment, the patient was advised to use a removable retainer called an Essix retainer for one year, and a regular follow-up was also advised. This retention phase was crucial for maintaining the achieved results and ensuring stability in the occlusal and esthetic corrections attained during the treatment period. By opting for a non-extraction approach and emphasizing the importance of diligent follow-up care with retainers, the treatment plan prioritized both short-term improvements and long-term stability, thereby addressing the patient's chief complaints while promoting optimal oral health and esthetic outcomes.

## Discussion

It has been reported that the most frequently seen feature of malocclusion is crowding [14]. Crowding or irregularly aligned teeth as stated by the patient occurs in the majority of the malocclusion patients [15]. Crowding affects the overall health of the oral cavity causing dental caries, periodontal problems, traumatic occlusion, etc., but among all of these, esthetics is something that every individual is concerned about in today's era. Crowding causes an unpleasant appearance for the patient while smiling. This reduces their confidence, which ultimately leads to psychosocial problems. This is the reason people look for orthodontic treatment [16,17]. Various methods by which crowding can be corrected are arch expansion transversely, interproximal stripping, posterior teeth distalization, anterior teeth proclination, and extraction. In patients where crowding is mild, about 0-4 mm and moderate about 4-8 mm [18], interproximal stripping is done, and extraction and arch expansion are standard treatment modalities [19-21].

In this case, we opted for a non-extraction approach for the alignment and leveling of the teeth. There are various cases where treatment of crowding is done without extraction. A case of severe crowding was treated with the help of a self-ligating appliance. These appliances help in the correction of crowding without extraction. The other advantages of these are there is minimum friction between the archwire and brackets, it allows longer intervals in between appointments, great patient comfort, and reduces the duration of treatment [22]. Another case was where there was extreme crowding in the upper and lower arch with the buccal eruption of both maxillary canines and lower left canines. Here, the non-extraction treatment approach was used by the distalization of molars using the pendulum appliance and continued by fixed appliance therapy plus proximal reduction [23]. Arch expansion can also be used as one of the treatment modalities for correction of crowding. This can be gained by the use of mini screw-assisted rapid maxillary expansion. This was used in a patient with severe mandibular crowding, mild maxillary crowding with retroclined upper incisors, severe deep bite, and overjet and Class II canine relationship [24].

Hence, it can be said that, in every crowding case, it is not always necessary to go for extraction. The abovementioned non-extraction approach can be used to obtain the best possible results.

## Conclusions

The case was diagnosed as Angle's Class I with Dewey's type 1 modification. The patient clinically presented with crowding in both the arches with complete deep bite. The malocclusion was treated with a non-extraction approach. The crowding in both the upper and lower arch was corrected. The convex profile was corrected to an almost straight profile, and the gummy smile was eliminated. A prefabricated edgewise appliance was used, followed by an SS wire for settling the occlusion. The posttreatment patient was advised to a regular follow-up plan with the use of a removable retainer over one year. There was slight crowding still present in mandibular anterior teeth at the end of the treatment as the patient wanted to discontinue the treatment because the family was moving to another city, but, overall, the patient was satisfied with the course of treatment and its outcome.

## References

[1] Tikar NN, Reche A, Jadhav V, Madhu PP, Chhabra KG, Dadgal KV, Pathekar JM (2021). Assessment of efficacy of novel method of quantifying and grading class II malocclusion in
vidarbha population. J Pharm Res Int.

[2] Shivakumar KM, Chandu GN, Subba Reddy VV, Shafiulla MD (2009). Prevalence of malocclusion and orthodontic treatment needs among middle and high school children of Davangere city, India by using dental aesthetic index. J Indian Soc Pedod Prev Dent.

[3] Quaglio CL, de Freitas KM, de Freitas MR, Janson G, Henriques JF (2011). Stability and relapse of maxillary anterior crowding treatment in class I and class II Division 1 malocclusions. Am J Orthod Dentofacial Orthop.

[4] Mageet AO (2016). Classification of skeletal and dental malocclusion: revisited. Stoma Edu J.

[5] Utari TR, Putri MK (2019). Orthodontic treatment needs in adolescents aged 13-15 years using orthodontic treatment needs indicators. J Dent Indones.

[6] Abela S (2013). An introduction to orthodontics. Eur J Orthod.

[7] Narmada IB, Ramadhani V, Pratiknjo IS, Prastiwi W (2023). Treatment of a patient with class I malocclusion with moderate crowding and missing first molar: a case report. Acta Med.

[8] Das PJ, Dkhar W, Pradhan A (2024). An evaluation of dental crowding in relation to the mesiodistal crown widths and arch dimensions in southern Indian population. J Clin Diagn Res.

[9] Cai B, Zhao XG, Xiang LS (2014). Orthodontic decompensation and correction of skeletal Class III malocclusion with gradual dentoalveolar remodeling in a growing patient. Am J Orthod Dentofacial Orthop.

[10] Ileri Z, Basciftci FA, Malkoc S, Ramoglu SI (2012). Comparison of the outcomes of the lower incisor extraction, premolar extraction and non-extraction treatments. Eur J Orthod.

[11] Garcia R (1985). Leveling the curve of Spee: a new prediction formula. J Charles H. Tweed Int Found.

[12] Germane N, Lindauer SJ, Rubenstein LK, Revere JH, Isaacson RJ (1991). Increase in arch perimeter due to orthodontic expansion. Am J Orthod Dentofacial Orthop.

[13] Kapadia RM, Shah AP, Diyora SD, Rathva VJ (2014). Non-surgical treatment of skeletal class III malocclusion. Non-surgical treatment of skeletal Class III malocclusion. Case Reports.

[14] Alam M, Nowrin S, Shahid F (2018). Treatment of angle class I malocclusion with severe crowding by extraction of four premolars: a case report. Bangladesh Med J.

[15] Turk T (2013). Orthodontics: principles and practice. https://onlinelibrary.wiley.com/doi/book/10.1002/9781118785041.

[16] Bittencourt MA, Farias AC, Barbosa MD (2012). Conservative treatment of a class I malocclusion with 12 mm overjet, overbite and severe mandibular crowding. Dental Press J Orthod.

[17] Jaswani M, Jaiswal P, Reche A (2023). Wilckodontics: a multidisciplinary approach for orthodontic treatment. Cureus.

[18] Lapenaite E, Lopatiene K (2016). Interproximal enamel reduction as a part of orthodontic treatment. Stomatologija.

[19] Pindoria J, Fleming PS, Sharma PK (2016). Inter-proximal enamel reduction in contemporary orthodontics. Br Dent J.

[20] Luppanapornlarp S, Johnston LE Jr (1993). The effects of premolar-extraction: a long-term comparison of outcomes in "clear-cut" extraction and nonextraction Class II patients. Angle Orthod.

[21] Howe RP, McNamara JA, O’Connor KA (1983). An examination of dental crowding and its relationship to tooth size and arch dimension. Am J Orthod.

[22] De Lima DV, Freitas KM, De Freitas MR (2024). Nonextraction treatment of severe crowding with a self-ligating appliance. J Clin Orthod.

[23] Jain A, Jain G, Bhide M, Sharma P, Sapre J, Ringane A (2021). Non-extraction orthodontic treatment in a patient with severe anterior crowding. J Oral Med Oral Surg Oral Pathol Oral Radiol.

[24] Altamash S, Sakrani H, Ahmed N, Marya A, Heboyan A (2022). Non-extraction orthodontic treatment for severe dental crowding using miniscrew-assisted rapid maxillary expansion. J Surg Case Rep.

